# Erratum

**Published:** 2014-10-03

## Vol. 61, No. 53

In the *MMWR* report “Summary of Notifiable Diseases — United States, 2012,” the Internet link on page 2, second column, line four was incorrect. It should read as follows: **http://wwwn.cdc.gov/nndss/script/history.aspx**.

